# Detection of *Aedes flavopictus* (Yamada, 1921), Netherlands, June 2019

**DOI:** 10.2807/1560-7917.ES.2019.24.30.1900433

**Published:** 2019-07-25

**Authors:** Adolfo Ibáñez-Justicia, Bart van de Vossenberg, Rens van den Biggelaar, Joris Voogd, Eveline Metz, Frans Jacobs, Marian Dik, Arjan Stroo

**Affiliations:** 1 Centre for Monitoring of Vectors, National Reference Centre, Netherlands Food and Consumer Product Safety Authority, Wageningen, Netherlands; 2 Molecular Biology Group, National Reference Centre, Netherlands Food and Consumer Product Safety Authority, Wageningen, Netherlands

**Keywords:** Aedes flavopictus, the Netherlands, invasive mosquitoes, public health, surveillance, vector

## Abstract

In June 2019, a single specimen collected at a used tyre company was identified as *Aedes flavopictus* (Yamada, 1921), a sibling species of *Ae. albopictus*. *Ae. flavopictus* has not been recorded outside Japan and South Korea. Although it has only shown dengue virus vector competence under laboratory conditions, its detection demonstrates the value of active surveillance at risk locations and molecular tools for timely intervention against exotic mosquitoes with potential future public health impact.

On 13 June 2019, in a sample of mosquitoes captured in the province of Flevoland, the Netherlands during routine surveillance, a specimen was recognised as potentially belonging to an exotic species. Based on the molecular analysis of the specimen, we concluded that it belonged to *Aedes flavopictus* (Yamada, 1921). To our knowledge, this is both the first time that this species has been reported outside its area of origin in north-east Asia and associated with the import of used tyres.

## Mosquito surveillance at high-risk locations

From 2010 onwards, an annual quantitative risk assessment of all used tyre companies known to import tyres into the Netherlands is performed by the Centre for Monitoring of Vectors (CMV) of the National Reference Centre (NRC) of the Netherlands Food and Consumer Product Safety Authority (NVWA) for the purpose of assessing the risk of invasive mosquito species (IMS) introduction. Sampling methods and frequency of the inspections depend on the expected risk of that location [1]. A company in the province of Flevoland has been categorised as high risk based on earlier introductions of *Ae. albopictus* and their international trade with companies in areas where IMS are established. Consequently, mosquito trap sampling is carried out every 2 weeks during the period in which mosquitoes in the Netherlands are most active, i.e. from mid-April to mid-October. At this location, at least one BG-Sentinel trap lured with BG-Lure (Biogents, Regensburg, Germany) and CO_2_ is collecting mosquito specimens 24 hours a day, 7 days a week. Samples collected from used tyre companies are labelled in the field with a unique code, sealed and sent to the vector specialists of the CMV for further analysis.

## Detection of *Aedes flavopictus*

At the laboratory, mosquitoes are counted and morphologically identified using the electronic key of Schaffner et al. [2] and the key of Becker et al. [3]. Samples containing only indigenous mosquito species are considered negative samples and samples containing IMS are considered positive samples. In one sample of mosquitoes captured during surveillance at the tyre company in Flevoland, one specimen was recognised as a potentially exotic species. This sample was obtained from the BG-Sentinel trap within 28 May 2019 and 12 June 2019. The sample also contained 138 indigenous *Culex* spp. mosquitoes. All mosquitos collected were females. The potential exotic specimen was damaged and we were not able to reliably morphologically characterise it to the species level. As it was recognised as an *Aedes* species, one leg of the specimen was subjected to molecular identification using four real-time PCRs specifically developed for identification of *Ae. albopictus*, *Ae. aegypti*, *Ae. atropalpus* and *Ae. japonicus* [4]. All four simplex assays produced negative results and the DNA extracted from the specimen was further subjected to sequencing of the partial mitochondrial *cox1* gene (GenBank accession number MN102102), also referred to as DNA barcoding [5]. Blast analysis of the obtained 658 nt sequence in the Barcode of Life Data System version 4 using the species level barcode records for animal identification (BOLD Systems, Guelph, Canada) and the National Center for Biotechnology Information’s (NCBI) GenBank showed 98% coverage and 99.4% pairwise identity with an accession submitted by Maekawa et al. 2016 (GenBank accession number LC054355.1) [6]. Clustering of sequence MN102102 with the blast hits from BOLD Systems and NCBI showed grouping of the sequence in an *Ae. flavopictus* species-specific clade. Following the finding of this species, the trap was checked at the site during two additional sampling periods, 12–19 June 2019 and 19–26 June 2019, but only indigenous *Culex* spp. were identified.

## Climate suitability assessment

For a rapid assessment of the suitability of the Dutch climate for *Ae. flavopictus*, we used the Match Climates function of the CLIMEX software version 3 (Hearne Scientific Software, Melbourne, Australia). This function is a tool for comparing the meteorological data of different places without reference to any particular species. This function allows us to select a location, the Home location, and find other locations, the ‘Away’ locations, with climates similar to that of the ‘Home’ location. In CLIMEX, the similarity of the climate is measured by the Composite Match Index (CMI), a value between zero and one, with higher values corresponding to a greater match between locations [7]. For our study, we used two ‘Home’ locations: (i) Miyako, the nearest climate station to the location of the specimen that showed 99.4% sequence homology with our specimen (latitude/longitude = 40.8523 N/140.5310 E, North of Aomori province in Japan), and (ii) Sapporo, which is on the island of Hokkaido, where the species is known to occur (BOLD accession number GBDCU179–12). Results of the match climates function projected to Europe showed that the CMI values of several locations in the Netherlands, Amsterdam, Rotterdam and Groningen were around 0.7. In CLIMEX, a CMI > 0.7 means good matching. It is anticipated that winter temperatures in the Netherlands would not likely be a limiting factor for the species since the averaged minimum temperatures in Sapporo, Hokkaido, Japan are lower than in the Netherlands. This preliminary assessment shows that the climatological conditions are unlikely to be a limiting factor for *Ae. flavopictus* in the Netherlands or other temperate areas in Europe. Other ecological restraints that limit the invasiveness of this species might apply, so further studies are necessary.

## Control measures

The origin of the found specimen is currently unknown, and for this reason, the NVWA is performing an intensive survey to find its source.

Notwithstanding the relative low demonstrated risk for public health, the Dutch authorities have put a plan to prevent possible spread and establishment of the species into place. First, the complete company site is being treated by spraying *Bacillus thuringiensis israelensis* (Bti) (VectoBac WG, Valent BioSciences, Libertyville, Illinois, United States) against larvae inside the tyre stacks stored outside and deltamethrin (dilution rate of 2.5 ml/100 ml water) (Aqua K-Othrine, Bayer Environmental Science, Leverkusen, Germany) against adult mosquitoes. Second, larval control of the surrounding area, a predefined perimeter of 500 m, consists of removing potential larval breeding sites for container-breeding *Aedes* spp., or treating breeding sites with either aqueous spray of Bti (VectoBac WG) or Bti/*Bacillus sphaericus* (Bs) granules (VectoMax FG, Valent BioSciences). Larviciding is being repeated every 2 to 3 weeks until the end of October, which is when ambient conditions for IMS become generally unfavourable. Currently, there is no information of any resistance of this species to the above mentioned biocides.

## Discussion

In this article, we report to our best knowledge, the first finding of the species *Ae. flavopictus* outside its area of origin, and the possible association of this finding with the import of used tyres. It is unlikely that the specimen found in the Netherlands was imported from other European countries since, to our best knowledge, the species has not been reported in Europe previously. Preliminary results of inquiries at the company confirms that it regularly imports and stores tyres from Japan. This makes it plausible that there was passive transport of *Ae. flavopictus* eggs through the import of used tyres from the area of origin in Japan.

*Ae. flavopictus* is widely distributed in Japan and South Korea and comprises three subspecies: *Ae. flavopictus* in the Palearctic region of Japan, *Ae. flavopictus downsi* from the Amami and Okinawa islands, and *Ae. flavopictus* *miyarai* from Ishigaki Island and Iriomote Island of the Ryukyu archipelago [8]. *Ae. flavopictus* has been described as a rare species in these countries [9], confined to forests and its periphery [10] where females of the species are considered severe day biters [8]. However, the results of Chaves analyses [9] suggest that *Ae. flavopictus* might be antagonistically interacting with *Ae. albopictus* in Japan and spreading because of its ability to cope with the variability of changing environments. This analysis suggests the emergence of this species as a new dominant vector species in communities currently associated with known disease vectors such as *Ae. albopictus*. It is important to note that *Ae. flavopictus* has a similar morphology to that of other *Aedes* species [11], including to that of *Ae. albopictus*, and that both species co-occur, occupying similar breeding sites in Japan [9].

In comparison with *Ae. albopictus*, *Ae. flavopictus* is not known to be a vector of infectious diseases in the field. However, under laboratory conditions, the species has been shown to be a competent vector of dengue [12,13]. Besides dengue virus, *Ae. flavopictus* has been found infected in Japan with *Aedes* flavivirus (AEFV), an insect-specific flavivirus that does not seem to infect vertebrates and that replicates exclusively in mosquito derived cell lines [14].

The international transport of used tyres leads to exotic and potentially invasive mosquito species being introduced to non-endemic areas where they may have the potential to reproduce and establish. The introduction of IMS with potential to transmit vector-borne diseases are of particular concern. While *Ae. flavopictus* is not known to be a vector of infectious diseases in the field, its detection in Europe is worth noting as it raises awareness of the possibility of new species with different vector competence being introduced via known pathways. Its detection also demonstrates the need for proper identification of morphologically similar mosquito species with different vector competence, e.g. *Ae. albopictus, Ae. cretinus* and *Ae. flavopictus,* or *Ae. japonicus* and *Ae. koreicus*, and underlines the importance of active mosquito surveillance systems for timely intervention against exotic mosquitoes with potential future public health impact.
